# QUANTIFYING INTRINSIC HEALTH USING STRESS-EVOKED INFORMATION FLOW BETWEEN PHYSIOLOGICAL SYSTEMS

**DOI:** 10.1093/geroni/igad104.0117

**Published:** 2023-12-21

**Authors:** Yan Wang, Sze Pui Tsang, Zain Khan, Daniel Malinsky, Ying Wei, Alan Cohen, Martin Picard, Sen Pei

**Affiliations:** Columbia University, New York City, New York, United States; Columbia University, New York City, New York, United States; Columbia University, New York City, New York, United States; Columbia University, New York City, New York, United States; Columbia University, New York City, New York, United States; Columbia University, New York City, New York, United States; Columbia University Irving Medical Center, New York City, New York, United States; Columbia University, New York City, New York, United States

## Abstract

As a canonical complex dynamical system, human bodies require interactions between constituent physiological systems to perform functions and maintain health status. Quantifying the dynamic interactions between organ systems using biomarkers collected in the context of psychobiological challenges may provide a novel means of measuring the energy-dependent communication processes that underlie intrinsic health. In this study, we use transfer entropy (TE), an information-theoretic metric, to quantify information flow between three biomarkers (heart rate, blood pressure, and respiration) in 71 participants of the Mitochondrial Stress, Brain Imaging, and Epigenetics (MiSBIE) project. In this stress experiment, both healthy individuals (n=44) and participants with genetic mitochondrial disorders (Mutation n=16, Deletion n=11) were exposed to a standardized 5-minute socioevaluative challenge (i.e., public speaking). Heart rate, blood pressure, and ventilation were collected continuously across four phases (baseline, pre-task, during the challenge, and post-challenge) over a 2-hour period. Using time series constructed from the three biomarkers, we compute six pairwise TE values for each participant in each phase, measuring the direction and strength of the information flow between physiological systems. Across different phases, we identify significant differences in TE values between healthy participants and patients with mitochondrial diseases. For example, during the post-challenge phase, TE from blood pressure to respiration in healthy participants was 19% higher than the deletion group (p=0.038). Our finding highlights the potential use of TE as a computational tool for detecting mitochondrial disorders and, more broadly, quantifying intrinsic health using information transfer between physiological biomarkers.

